# Teaching and Learning Modalities in Higher Education During the Pandemic: Responses to Coronavirus Disease 2019 From Spain

**DOI:** 10.3389/fpsyg.2021.648592

**Published:** 2021-08-24

**Authors:** Ana Verde, Jose Manuel Valero

**Affiliations:** ^1^Faculty of Law and Social Sciences, King Juan Carlos University, Madrid, Spain; ^2^Faculty of Education, International University of La Rioja, Logroño, Spain; ^3^Faculty of Health Sciences, King Juan Carlos University, Madrid, Spain; ^4^Faculty of Education, Camilo José Cela University, Madrid, Spain

**Keywords:** teaching and learning modalities, educational technology, COVID response, performance, satisfaction, ICT, teacher competencies, higher education

## Abstract

The effects of the pandemic have affected and continue to affect education methods every day. The education methods are not immune to the pandemic periods we are facing, so teachers must know how to adapt their methods in such a way that teaching, and its quality, is not negatively affected. This study provides an overview of different types of teaching methodology before, during, and after the coronavirus disease 2019 (COVID-19) pandemic. This study describes the different types of teaching (e.g., presence learning, blended learning, and distance education) used in two Spanish Universities (i.e., one private and one public) during the pandemic. A new teaching methodology is proposed. The purpose of this study report is to share what we learned about the response to COVID-19. Results provide a basis for reflection about the pros and cons of teaching and learning modalities in higher education. The current situation demands that we continue to rethink what is the best methodology for teaching so that the education of students is not affected in any way. This study is useful for learning about different teaching methods that exist and which ones may suit us best depending on the context, situation, and needs of our students.

## Introduction

As a result of the situation caused by the State of Alarm driven by the coronavirus disease 2019 (COVID-19), the educational system has been forced to adapt to the new capacity requirements and, in many cases, cease their usual activity.

The Community of Madrid, Spain, forced the closure of educational centers on March 12, 2020. A few days later, on March 14, 2020, the State of Alarm was decreed for the entire Spanish territory for an initial period of 15 days with strict measures of confinement and with restrictions on the movement of people and on the economic activity. This confinement was extended until June 21, 2020. Thus, the longest State of Alarm in the history of Spain ended after 3 months of confinement to stop the spread of COVID-19, and the so-called “New Normal” began. The restrictions on the movement between the Spanish provinces ended, and the coexistence with the virus began.

One of the most important decisions made at the educational level took place on April 14, 2020. The Government and the Autonomous Communities of Spain agreed that the academic year of 2019–2020 Educational System would end in June, and repetition would be exceptional at primary and secondary levels. Face-to-face classes, in general, would resume in September. During the 2019–2020 course, only those students who needed reinforcement or changed their educational stage, as well as children from 0 to 6 years old whose parents did not do telework, voluntarily returned to the classrooms. This was a very important shock not only for University levels but also for all educational levels.

From March to September 2020, due to the declaration of a State of Alarm by the National Government, the educational centers could not be opened, and they had to optimally adapt to this fact. Each educational center had to base its teaching on the online mode and to adapt teachers and students to this new reality: videoconferencing software was used to avoid social disconnection, students were disoriented, ignorance of new tools had to be overcome to teach classes, and the evaluation systems need to be redesigned. The pandemic revealed the shortcomings of educational institutions, mainly about the infrastructures and the training of teachers in the Information and Communication Technology (ICT) tools. However, it also meant improvements. The teachers were trained in new online methodologies and showed interest in learning new teaching tools in the face of the new reality and challenges that arose.

As of the new academic year 2020–2021, which began in September 2020, this teaching modality became eligible again. Each University, therefore, chose the type of methodology that it would use to carry out in its classes. In the universities themselves, depending on the faculties and the studies, different teaching methodologies are currently used.

## Effects of COVID-19 on the Education Performance of University Students in Classrooms

**Figure 1:**
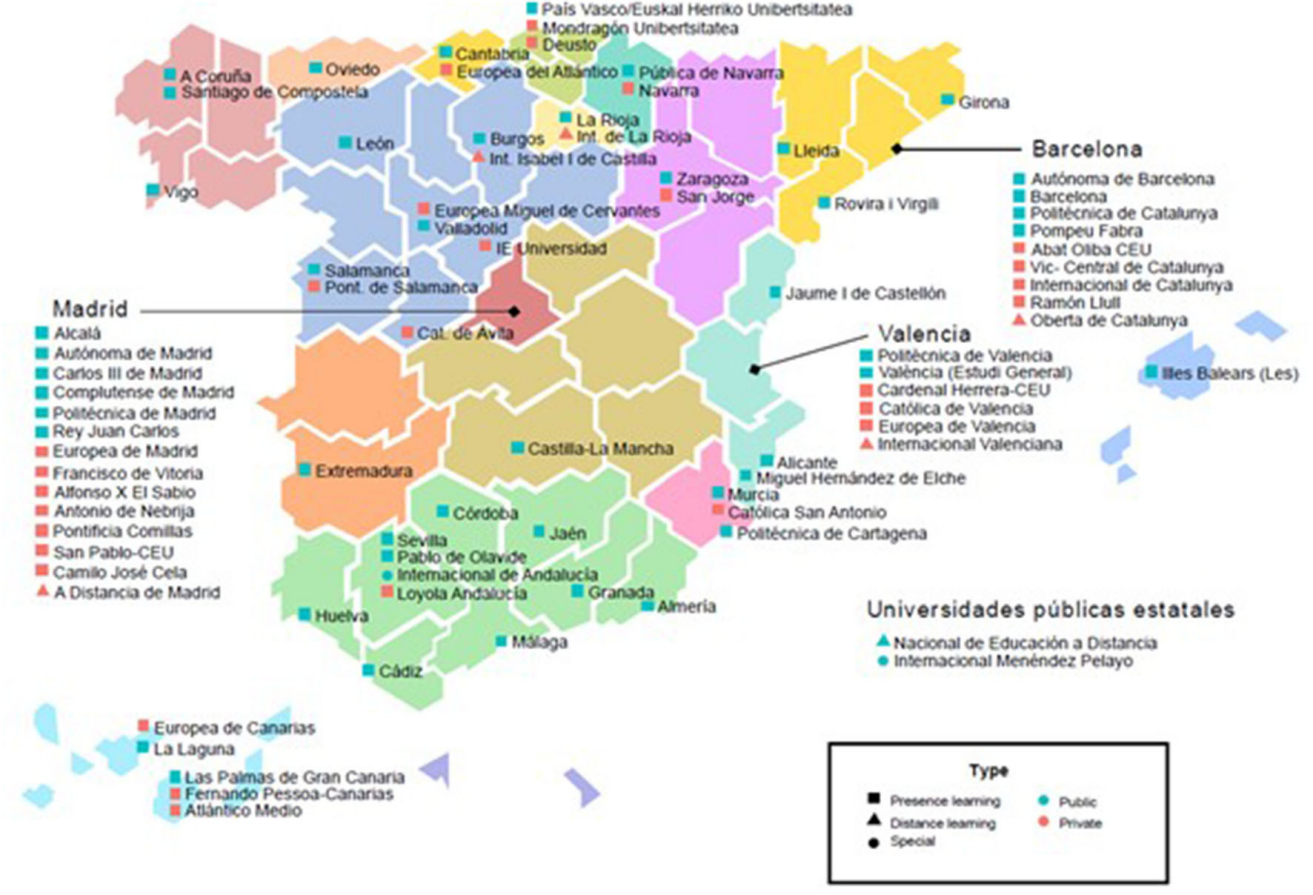
Geographical distribution of the Spanish universities with activity in the academic year 2019–2020. Statistics report (2020–2021) from the Ministry of Education and the Ministry of Universities. Universidades.gob.es. 2021. Available online at: https://www.universidades.gob.es/.

The Spanish University System (SUE) is made up of a total of 83 universities−50 public and 33 private (Figure 1).

- Out of the 50 public universities, 47 offer presence learning, and 1 offers distance learning.
- Out of the 33 private universities, 28 offer presence learning, and 5 offer distance learning.

In addition, there are two public universities with a special status that provide only specialized postgraduate programs (master's and PhD courses).

Factors considered in the following sections are the type of University, whether it is public or private, and the type of studies pursued, since there will be some in which an online model has already been implemented naturally, or vice versa, i.e., 100% face-to-face modality. As of September 2020, Spanish universities have used the teaching methods detailed in the following sections.

### Presence Learning

*Presence learning* consists of both the students and the teacher sharing the same physical classroom. Previous studies have emphasized the educational benefits of the use of this teaching practice (Bigg, 2003; Konopka et al., 2015; Crisol Moya, 2016; Anderton et al., 2021; García-Peñalvo et al., 2021). This type of teaching methodology could not be applied from March to September 2020 due to the declaration of a State of Alarm by the government of the nation. However, as of September 2020, this teaching modality became eligible, and the educational centers were reopened.

The non-face-to-face teaching model is becoming increasingly popular in the field of higher education. Universities traditionally oriented to face-to-face teaching, regardless of whether they are public or private, are embracing this model. Although they maintain their main face-to-face structure, they offer students some distance-based degrees and master's studies (Ben-Chayim and Offir, 2019; Ali, 2020; Hodges et al., 2020).

A face-to-face University that decides to include non-face-to-face teachings in its degrees and master's studies must combine its traditional procedures with the new requirements of non-face-to-face teaching (Chick et al., 2020). The universities that have already had this experience, even though they have been mostly presential, have been able to adapt more quickly to the suspension of in-person activity.

### Distance Education

Distance education, also known as online learning, is a type of education developed using technology that allows students to attend classes in remote locations (Dede, 1990; Hodges et al., 2020; Sandars et al., 2020). It can also be defined as a type of education that joins professors and students from different locations. Although they maintain their main face-to-face structure, they offer students some distance-based degrees and master's studies. On the one hand, several authors have recognized that online teaching can be synchronous when the students and the teacher connect to the classes at the same time and can have real-time interactions. On the other hand, in asynchronous teaching, the teacher and the students do not have to coincide in the class. Usually, the class is recorded, and the students can view it at any time (Adedoyin and Soykan, 2020; Ali, 2020; Bao, 2020).

This type of teaching, which was already followed before the pandemic, was not affected by the pandemic (Hwang, 2018; Daniel, 2020). Distance education is characterized by having an existing organizational infrastructure, which allows the educational objectives of online learning to be developed (Singh and Hardaker, 2014).

We must not confuse this type of teaching, i.e., distance education, with the *Emergency remote education*. In exceptional situations that impede the normal functioning of institutions and face-to-face educational centers, teachers may be forced to quickly adapt their pedagogical activity to a virtual environment. This is known here as emergency non-face-to-face teaching. In Spain, from March to June 2020, all teaching methods were entirely online. The emergency remote teaching required by the pandemic was often quickly improvised, without guaranteed or adequate infrastructure support (Evans et al., 2020; Hodges et al., 2020; Panisoara et al., 2020). Given this lack of infrastructure, the main source of advice and early support for non-expert distance teachers was focused on providing the technological tools available in each institution and was considered adequate to support the change.

### Blended Learning

This model is based on a combination of classroom education and online education in various forms (Lightner and Lightner-Laws, 2016; Nuruzzaman, 2016; Heilporn et al., 2021). There is no unanimity of criteria, since the meaning is ambiguous, causing confusion, and gives rise to a certain lack of rigor between the different types of blended learning (Misseyanni et al., 2018; Bao, 2020). It is necessary to distinguish between hybrid teaching, mirror classrooms, blended teaching, and the new methodology proposed in this study, i.e., online guides in the classroom.

#### Hybrid Learning

*Hybrid education* assumes that half of the students in a class attend the classroom and the other half follow the class from home, partially online and partially face-to-face (Misseyanni et al., 2018; Bao, 2020).

The use of the *hybrid-flexible* (*HyFlex*) instructional methodologies is relatively recent in higher education (Beatty, 2019). As has been previously reported in descriptive case studies, the *HyFlex* techniques are implemented by an instructor. Previous research has shown efforts to include this methodology, although few studies report the impact on student learning and the associated metrics of interest, such as qualifications, retention, pass rate, and time to graduation (Lightner and Lightner-Laws, 2016; Beatty, 2019, Binnewies and Wang, 2019; Mumford and Dikilitaş, 2020).

From September 2020, in Spanish universities that followed this methodology, groups of face-to-face students and online students alternated to achieve social distance without having to modify the structure of the classrooms.

#### Mirror Rooms

With the accumulated incidence of COVID-19, one of the options used in Spanish University education was the so-called “*Mirror Rooms*,” which allows face-to-face classes but at a safe distance, ensuring a distance of at least 1.5 m between the chairs. To maintain the safety distance measures in the case of not having large enough classrooms, the group of students is divided into two subgroups. Half of the group is in a classroom, with the teacher, while the other half is in an adjoining classroom, watching the class by live videoconference. The advantage of this typology compared with hybrid education, in which half of the students follow the class from home, or compared with a blended education, in which the face-to-face education is alternated with online teaching, is that, in *Mirror Rooms*, the students do not depend on their resources or the connection in their homes, since the entire process is carried out in the educational center, including the online part. They have their classmates in class for support and motivation and are able to continue enjoying contact with classmates and a University environment (Misseyanni et al., 2018).

The drawbacks of this type of methodology are the need to have enough classrooms, in addition to the technical resources necessary to broadcast the class live and personnel who can control these mirror classrooms. Another disadvantage supposes students do not have any engagement directly with the lecturer, who will be in standing in another classroom, being a similar situation to that in asynchronous online classes. In University studies, this methodology may be feasible, but not so much in other educational stages, in which it will not be easy for students to be alone in a class and pay attention.

#### A “Semi-Presential Learning” Blended System

The approach of the blended system is mostly carried out with the alternation between face-to-face classes and online education, either by videoconference or by independently following, i.e., individually or in groups, the tasks begun in class in person. A variety of this model is splitting up students and having those groups take turns going to class. According to the study by Cândido, in the semi-present context, students alternate online activities with face-to-face meetings (Cândido et al., 2020). This means fewer contact hours for each subject, which will be compensated with work from home. For example, if we work on projects, students can take part in the classroom and stay at home when they cannot go to the school in person.

### Online Guide in the Classroom

Another new approach to blended learning education that is proposed in this study is what we have called the “*Online guide classroom*.” This new methodology has been made evident by the new reality of the pandemic. A person who has been in contact with another person who has tested positive for COVID-19 should take the contagion test and stay at their home until the results of the test are known. In this situation, teachers, who physically have no symptoms and are well, have noticed how their teaching has been interrupted, being a detriment to their students.

In an *Online guide classroom*, the teacher stays at home, or another location, and teaches through a computer, and the students physically travel to the campus to follow the video conference. The advantage of this type of teaching is that, if the teacher is in the previous situation, is in quarantine and might have been exposed to COVID-19, or is even unable to attend a class, e.g., for other activities, e.g., assisting a Congress in another country, students will not miss class. In addition, students will be able to continue enjoying University life and to carry out group work in person with their classmates. This option is particularly necessary for science students who need to work in the laboratories for their lessons (Anderton et al., 2021). The classroom will need to meet certain technical requirements to be able to project the videoconference, e.g., microphones and cameras incorporated in the classroom, i.e., the same hybrid-learning technical resources that prior research suggests (Hwang, 2018; Bao, 2020; Salikhova et al., 2020), as well as staff or students responsible for connecting these devices.

## Methodology

An investigation was carried out with the purpose of analyzing whether the change in the teaching–learning methodology, due to COVID-19, diminished in any way the quality of the education and/or the satisfaction of the students.

The sample consisted of 307 University students who voluntarily decided to participate in the investigation and who were subdivided into two groups. The first group, surveyed in November 2020, was made up of 152 University students, 128 women and 24 men between 19 and 22 years of age. The second group, surveyed in February 2021, was made up of 155 University students, 57 women and 98 men between 19 and 22 years of age, so the sample consisted of a total of 185 women and 122 men. The students pursued different University studies as follows: Early Childhood Education, Primary Education, and Physical Activity and Science Sports + Physiotherapy (a double degree program). We were especially interested in the point of view of the former two groups because they will become teachers. We added students not directly related to education to the sample for more heterogeneity. In addition, the first group of students, surveyed in 2020, was composed of students from a private University, and the second group, surveyed in 2021, was composed of students from a public University. The percentage of women in the first group of students surveyed was much higher, but this was compensated with the incorporation of the second group of students surveyed, reaching a final proportion of 60% women and 40% men.

To conduct the study, the surveys were sent to each student through Google Forms to avoid paper processing and to facilitate their completion, each of them having been previously informed of the study objectives.

Before administering the questionnaire to the students, compliance with all the required ethical standards was ensured as follows: written informed consent, the right to information, confidentiality, anonymity, gratuity, and the option to abandon the study (MacMillan and Schumacher, 2001).

This research was not approved by an ethics committee, since the data are not clinical or sensitive, although they were anonymized.

All questions used for our study referred to three possible classroom situations experienced by all students from 2020 to 2021. The study was carried out in the different modalities developed according to the governmental restrictions in Spain and around the world as follows:

- First: The pre-COVID-19 scenario, without any social restriction: a face-to-face environment. Location(s): University.
- Second: The COVID-19 scenario, with all social restrictions: a confinement situation, and the perimetral lockdowns of regions: an online environment. Location(s): Home.
- Third: The current scenario, the COVID-19 scenario with some restrictions, living with the virus: a combination of online, semi-presential, and online guide classrooms. Location(s): Home and University.

Throughout the academic year 2020–2021, no student was able to attend their classes in the same way they did at the beginning of the previous year, since the pandemic was still in force; however, in our study, all students surveyed had experienced all scenarios because they were all University students who were currently in their second year of University studies. Therefore, the two groups surveyed were able to answer the study questions based on their experience of classes without restrictions in the previous year, before the pandemic broke out, and to compare that with the current restrictions.

Students, as well as the teacher, had to adapt to the situation that was being experienced around the world and to change their teaching–learning methodology in order to continue learning. The main objective since the beginning of all these changes was to preserve the natural progress of the classes and ensure that the changes in methodology did not affect the quality of the education and the satisfaction of the students.

To analyze whether this objective was being achieved, students were analyzed in regard to their satisfaction, the quality of the education, and the feelings of the students during these new conditions and education modalities.

### Instrument

Two instruments were used to measure educational quality and student satisfaction in regard to the three educational modalities described earlier. Six main items were assessed, in addition to the academic quality and the satisfaction of the students, over the three phases listed earlier. In the case of the 6 items, students were given a questionnaire made up of 6 parameters to be assessed individually. Each participant responded using a 10-point Likert-type scale, 1 being “totally disagree” and 10 “totally agree.”

Items studied were as follows:

1. Accessibility
2. Satisfaction
3. Participation
4. Results obtained
5. Innovative value of teaching practice
6. Information and Communication Technology (ICT) Knowledge.

In the case of academic satisfaction with the classes, the scale suggested by Lent et al., composed of seven items that assess the degree of the satisfaction of students with respect to academic activity, was used (Lent et al., 2005). Each of the students who participated in the study responded using a 5-point Likert-type scale, 1 for “totally disagree” and 5 for “totally agree.”

### Limitations

The approach utilized suffers from the limitation that the study only captured the sample of two Spanish universities, i.e., one private and one public. The degrees studied are related to the field of Education (75%) and Science (25%): a degree in Early Childhood Education, a degree in Primary Education, and a degree in Physical Activity and Sports + Physiotherapy.

### Statistical Analysis

Using the SPSS (IBM SPSS Statistics) statistical software, the missing values were evaluated, considering the items of each instrument to estimate whether it responded to a random distribution (Tabachnick et al., 2011). The arithmetic mean, SD, asymmetry, and kurtosis were also calculated in each case. As a criterion to evaluate the asymmetry and kurtosis indices, values between −1.00 and 1.00 were considered excellent, and values within the range of −2.00 to 2.00 were considered adequate (George and Mallery, 2011).

## Results

Compliance with the statistical assumptions was verified, the exploratory factor analysis was applied to demonstrate the underlying structure of the scale, and its internal consistency was estimated using the Cronbach's alpha statistic. First, the amount and pattern of the missing data were examined using the Missing Value Analysis Routine in SPSS. Since no variables that present more than 5% of the missing values were observed, no studies were conducted to evaluate the randomness pattern of the missing values. No outliers were observed (Goodyear, 2020). Following the recommendations of Zabala, the *t* distribution was used to determine the statistical significance, and the favorable results were obtained without having any case that exceeded the threshold considered (Zabala and Arnau, 2015).

The descriptive statistical values of the arithmetic mean and SD were calculated, and the asymmetry and kurtosis indices were obtained to analyze the normality of the distributions. Asymmetry and kurtosis indicated the shape of the distribution of our variables. These measurements allowed us to determine the characteristics of their asymmetry and homogeneity without the need to represent them graphically. All the variables presented indices between −1.5 and 1.5. Results were considered optimal for carrying out the planned statistical analyses (George and Mallery, 2011).

Three studies were carried out with the sample collected: Study 1 corresponds to the first group surveyed in November 2020, Study 2 corresponds to the second group surveyed in February 2021, and Study 3 corresponds to the entire sample, i.e., the first and second groups.

### Study 1

The average evaluation of the *presence learning* was 7.2, with satisfaction and participation being the highest-valued items in this modality. Knowledge in ICT was the least necessary for performance.

In the case of the *online modality*, we observed the highest scores in accessibility and knowledge in ICT but the worst score in participation. In this case, an average of 7.8 was reached.

**Figure 2:**
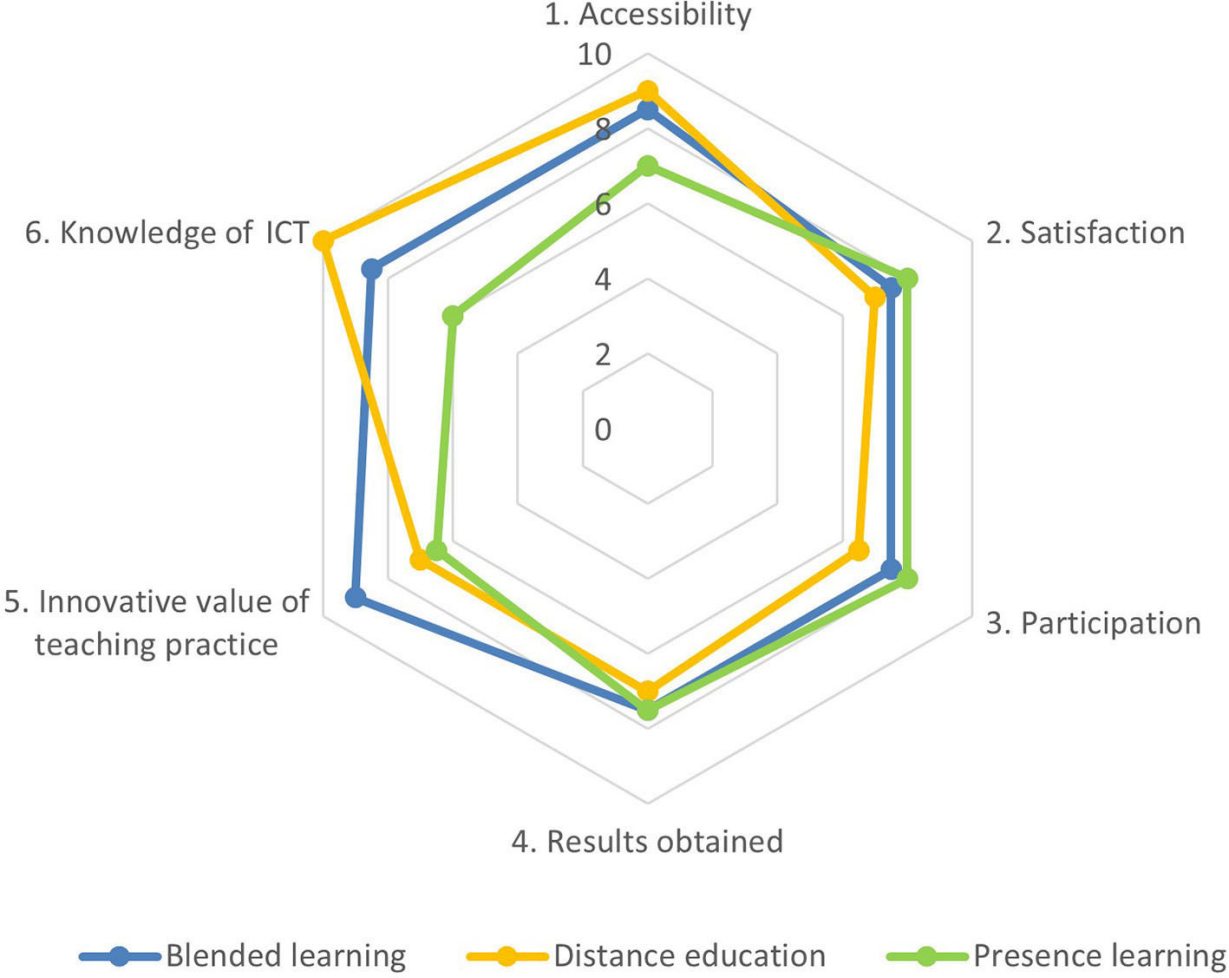
Comparison of items in Study 1. Source: Own elaboration.

The *blended learning modality* reached an average of 8.1, obtaining scores around 7.5 and 9.3. The innovative aspects of this modality were most highly evaluated (Figure 2).

If we use the asymmetry and kurtosis values, it can be affirmed that, in all items, there is a high degree of concentration around the arithmetic mean. All participants agreed on the positivity of all the modalities of the teaching–learning classes of the study. That is, all students considered the practice carried out to be excellent and highly beneficial for their education, and their satisfaction was not affected by the fact of having to change modalities during the course, although the students of this group, in general terms, preferred blended learning.

Regarding Table 1 on the *blended learning* from Study 1, the degree of satisfaction in the students was very high, which indicates an excellent degree of significance (George and Mallery, 2011). In addition, all variables have a high SD and high asymmetry and kurtosis indices. The innovative value of teaching practice was scored with a 9.3, the highest score.

**Table 1:** Descriptive statistical analysis of the Academic Activity Scale in Study 1 with blended learning.

**Items**	**Arithmetic mean**	**SD**	**Asymmetry**	**Kurtosis**
1	8,50	0,67	−0,85	−0,07
2	7,50	0,30	−0,19	−0,77
3	7,50	0,96	−0,44	−0,25
4	7,50	0,70	0,97	0,44
5	9,30	0,42	0,55	−0,27
6	8,50	0,86	0,09	0,89

*Source: Own elaboration*.

Table 2 represents the scores and indices of the statistical analysis of *distance education*. The best-scored item was Item 6, i.e., knowledge of ICTs reached 9.9 and had high asymmetry and kurtosis indices. The worst score was for the item that evaluated participation. Still, satisfaction reached a remarkable score.

**Table 2:** Descriptive statistical analysis of the Academic Activity Scale in Study 1 with distance education.

**Items**	**Arithmetic mean**	**SD**	**Asymmetry**	**Kurtosis**
1	9,00	0,49	−0,11	−0,77
2	7,00	0,35	−0,27	1,20
3	6,50	0,14	0,98	−0,06
4	7,00	0,07	1,22	1,64
5	7,00	0,85	0,77	−0,02
6	9,90	0,07	−0,72	−0,15

*Source: Own elaboration*.

Finally, Table 3 represents the scores and indices of the statistical analysis of *face-to-face education*. Participation and satisfaction were the best-rated items here, i.e., the best in the entire Study 1, and the worst was for knowledge about ICT applications.

**Table 3:** Descriptive statistical analysis of the Academic Activity Scale in Study 1 with presence learning.

**Items**	**Arithmetic mean**	**SD**	**Asymmetry**	**Kurtosis**
1	7,00	0,21	0,99	−0,68
2	8,00	0,78	0,52	−1,25
3	8,00	0,50	−0,35	−1,40
4	7,50	1,13	0,23	−1,49
5	6,50	0,85	0,75	0,11
6	6,00	0,57	0,34	0,16

*Source: Own elaboration*.

### Study 2

In this study, the mean scores of the three teaching modalities improved in most cases. The average evaluation of the *presence learning* is 8.8, with the second- and third-most valued items being satisfaction and accessibility.

In the case of the *online modality*, we again observed that the highest score was for knowledge of ICTs. The average evaluation in this case was 7.9.

**Figure 3:**
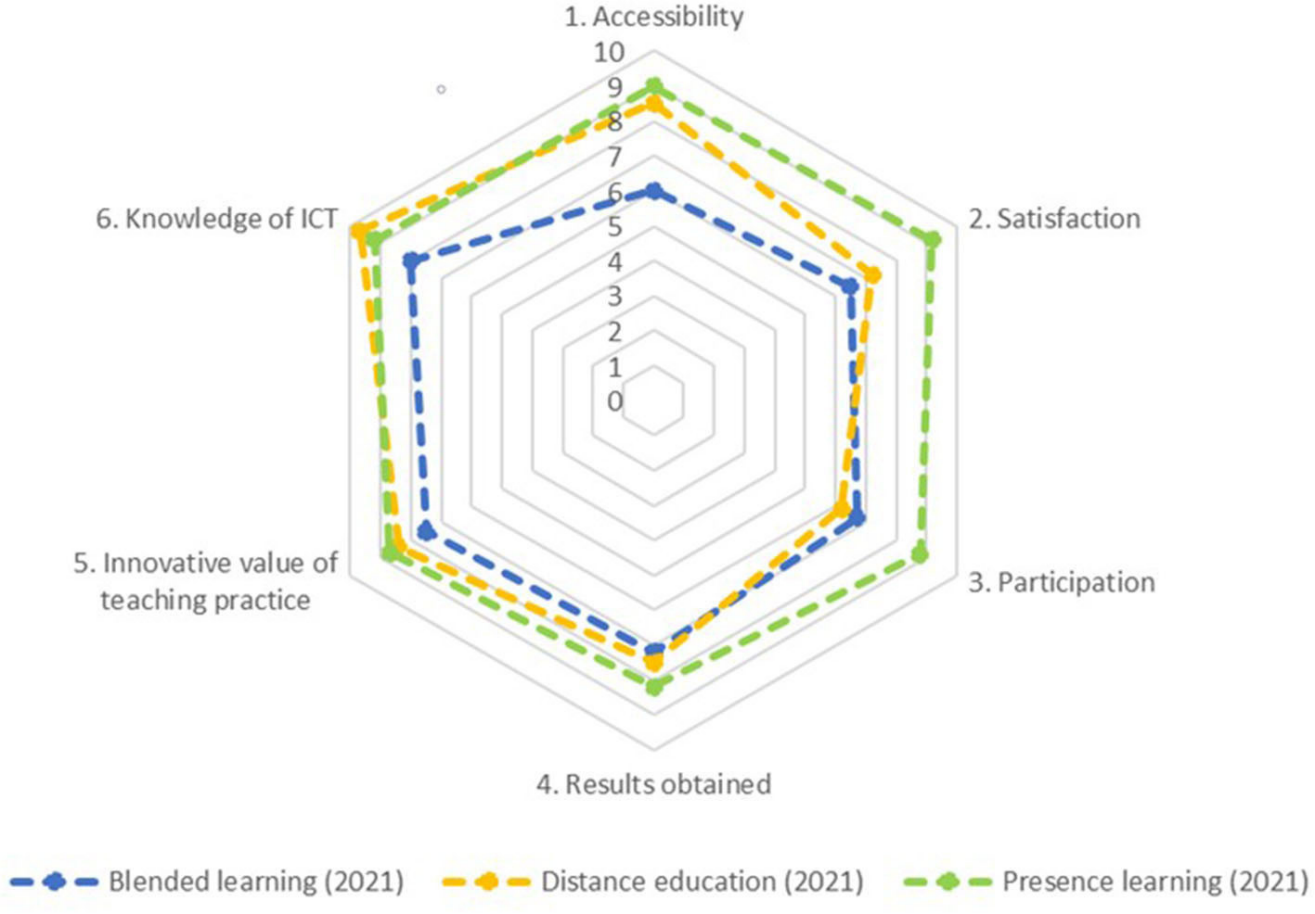
Comparison of items in Study 2. Source: Own elaboration.

The *blended learning modality* reached an average of 7.0, i.e., 1.1 percentage points lower than in the previous study. The best score was obtained for knowledge in ICTs, and the worst was for accessibility (Figure 3).

As in the previous study, the statistical values of the arithmetic mean, SD, asymmetry, and kurtosis were recalculated. All items obtained excellent or adequate scores depending on the teaching modality, but all of them are ideal, between −1.5 and 1.5, for studying the three teaching modalities (George and Mallery, 2011).

Regarding Table 4 on the *blended learning* from Study 2, the degree of ICT knowledge was high, but accessibility was scored with the worst value in this study for all learning modalities. Once again, it is remarkable that all the variables have ideal indices in terms of the arithmetic mean, SD, asymmetry, and kurtosis (George and Mallery, 2011).

**Table 4:** Descriptive statistical analysis of the Academic Activity Scale in Study 2 with blended learning.

**Items**	**Arithmetic mean**	**SD**	**Asymmetry**	**Kurtosis**
1	6,00	0,42	−0,04	−1,01
2	6,50	1,03	0,24	−1,20
3	6,70	0,52	0,72	0,20
4	7,20	0,99	−0,26	−0,41
5	7,50	1,30	0,34	−1,20
6	7,90	0,71	0,18	−0,34

*Source: Own elaboration*.

Table 5 represents *distance learning* and indicates once again that ICT knowledge has one of the best scores. The other scores are excellent, except for participation, which is once again the worst score for this teaching modality (6.2). There is great unanimity in this value due to the rest of the statistical indicators.

**Table 5:** Descriptive statistical analysis of the Academic Activity Scale in Study 2 with distance education.

**Items**	**Arithmetic mean**	**SD**	**Asymmetry**	**Kurtosis**
1	8,50	0,51	−0,08	−0,90
2	7,20	0,57	0,20	−0,04
3	6,20	0,08	0,72	0,11
4	7,50	0,57	0,68	−0,14
5	8,40	0,26	−0,89	0,52
6	9,70	0,21	−1,13	0,98

*Source: Own elaboration*.

Table 6 from Study 2 represents the *presence learning*, for which the scores of 9.2, 9.2, and 9.0 were obtained for satisfaction, ICT knowledge, and participation, respectively. The remaining scores for this modality were very high. The average score in this case, among all items, was 8.8, which means that it is the highest score achieved for any teaching modality among all of our studies.

**Table 6:** Descriptive statistical analysis of the Academic Activity Scale in Study 2 with presence learning.

**Items**	**Arithmetic mean**	**SD**	**Asymmetry**	**Kurtosis**
1	9,00	0,51	−0,23	−0,67
2	9,20	0,99	−1,32	0,99
3	8,80	0,08	−0,83	−0,88
4	8,20	0,57	0,28	−0,54
5	8,70	0,59	0,78	−0,67
6	9,20	0,07	−0,25	−1,46

*Source: Own elaboration*.

### Study 3

**Figure 4:**
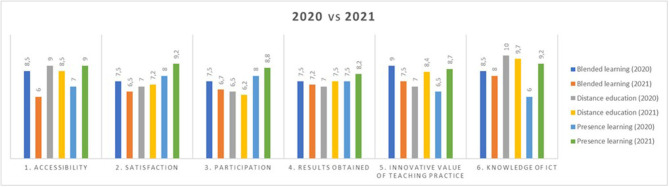
Result of the items studied “Comparative of teachings in bar diagram” in Study 3. Source: Own elaboration.

In Study 3, a comparison was made between Study 1 and Study 2, and a summary of both is provided. The following graph shows a comparison, one by one, of all items evaluated in the three teaching modalities in both studies (Figure 4).

The most notable evolutions are the 3.2-pp increase in ICT knowledge, the 2.2-pp increase in the innovative value of teaching practice, the 1.8-pp increase in participation, and the 1.2-pp increase in satisfaction with the *presence learning* in Study 2 compared with Study 1. These increases in scores will be explained in detail in the “Conclusion” section.

On the contrary, the greatest decrease in Study 2 compared with Study 1 occurred in accessibility in the *blended learning* modality, reaching a 2.5-pp decrease. Changes also occurred in the rest of the scores, but they were not as significant as the previous ones.

As shown in Tables 7–**9**, the complete sample is analyzed, i.e., Study Sample 1 and Study Sample 2, which composes the complete sample of Study 3.

**Table 7:** Descriptive statistical analysis of the Academic Activity Scale in Study 3 with blended learning.

**Items**	**Arithmetic mean**	**SD**	**Asymmetry**	**Kurtosis**
1	7,20	1,84	−0,01	−1,68
2	7,00	1,76	0,56	−0,26
3	7,10	0,62	0,02	−0,84
4	7,30	0,35	0,90	1,35
5	8,25	1,63	−0,10	−0,86
6	8,20	1,06	−0,38	0,11

*Source: Own elaboration*.

Table 7 shows the total scores for the entire sample for the *blended learning*. Its average score is 7.5, reaching its best scores in Items 5 and 6, where the innovative value is 8.25, and the value of ICT knowledge is 8.2, respectively. The worst score, i.e., the satisfaction of the students, is 7.0.

In Table 8, the complete *distance learning* sample is analyzed. The best score, as in previous studies, is the knowledge of ICT, with a score of 9.8. However, the worst score is the participation of the students (6.4), and excellent statistical indicators were obtained in all the statistical items. The average value for this modality is 7.8.

**Table 8:** Descriptive statistical analysis of the Academic Activity Scale in Study 3 with distance education.

**Items**	**Arithmetic mean**	**SD**	**Asymmetry**	**Kurtosis**
1	8,70	0,48	−0,24	−0,57
2	7,10	0,21	0,32	0,46
3	6,40	0,77	0,83	−0,01
4	7,25	0,42	0,86	0,29
5	7,70	0,30	−0,32	−1,22
6	9,80	0,07	−1,22	1,60

*Source: Own elaboration*.

As shown in Table 9 of our analysis on the *presence learning*, all statistical values are excellent and optimal (George and Mallery, 2011). The highest scores for satisfaction, participation, and accessibility stand out with 8.6, 8.4, and 8.0, respectively. In contrast, the values of innovation and ICT knowledge are each 7.6. Despite this, this modality obtains an average value of 8.0.

**Table 9:** Descriptive statistical analysis of the Academic Activity Scale in Study 3 with presence learning.

**Items**	**Arithmetic mean**	**SD**	**Asymmetry**	**Kurtosis**
1	8,00	1,85	−0,29	−1,49
2	8,60	0,92	−0,52	−1,28
3	8,40	0,86	−0,11	−1,07
4	7,90	0,42	0,14	−0,72
5	7,60	0,30	1,45	1,34
6	7,60	0,71	0,65	−0,97

*Source: Own elaboration*.

## Discussion

### The Pandemic, Teaching Methodologies, and Teaching Infrastructures

The pandemic has modified the vital context in which study plans are implemented for two reasons: First, the use of new platforms has been necessary, considering that the circumstances that have arisen require different methodologies from those used when the curriculum was originally designed. Second, both the knowledge and the professional competencies required to implement these methodologies are in the spotlight, requiring the training of professionals and students. The two universities studied, despite being *face-to-face universities*, have had different tools to quickly adapt to *Emergency remote teaching*. In both universities, while there are some online studies, professors who taught in the programs studied here had to become trained quickly because they were not professors in other online programs that continued to be taught without difficulty.

*Presence learning* involves contact with students, the elimination of technical difficulties, equal resources for all students, and the elimination of potential family conciliation problems for both teachers and students. In contrast, it has disadvantages as follows: the time needed to travel to the location of the class and the impossibility of connecting with students who live far from the educational center or even abroad. According to different authors, the change to online teaching has not meant a great change for many universities in the world (Ali, 2020). However, the transition to the large-scale online learning is a very difficult and complex task for education systems. As a UNESCO report indicates, even under the best circumstances, it has become a necessity (UNESCO, 2021).

*Online classes* are increasingly being held at prestigious International Universities both in America and Europe in which any subject can be carried out without face-to-face contact (Hwang, 2018). The idea behind this study, echoing the proposals of Bigg, is the recognition that any discourse on change and transformations of the school and its teachers is not limited only to the school environment. The participation of the educational community and, in this specific case of the University, the process of change can be the determining factors in the success of any innovation initiative that is attempted and, in the guarantee, through innovation and the use of ICT, of high levels of satisfaction in our students (Bigg, 2003; Tait, 2018; Bergdahl and Nouri, 2020; Reimers and Schleicher, 2020). The case of virtual teaching allows for more flexibility for learners who live in distant areas but also for teachers from different parts of the world. The platform used to broadcast classes live for the *Emergency remote education* (100% online or blended teaching) used in the private and public universities of this investigation was the same: Blackboard Collaborate. Blackboard Collaborate is a real-time video conferencing tool that allows one to add files, share applications, and use a virtual whiteboard to interact. One of the main advantages of Blackboard Collaborate is that one can run the application without having to install it on your computer. Blackboard opens directly in a browser without the need to install any software to join a session. The participation of the educational community and the involvement in the training of teachers in the two universities was evident. Both universities provided extensive help materials for the use of this tool, both written and in video format. In the case of the private University, a forum for interaction and the resolution of doubts was also set up. The private University provided an email to raise questions and gave live online courses for teachers.

The benefits of the *blended education* are for professors and students. With the potentiality of connection through the Internet, new learning possibilities arise, with added resources that help toward comfort, accessibility, effectiveness, and more options to access education. Classes in hybrid mode allow for optimization of the use of academic resources and grant control of the capacity and social distance, as there are fewer people in the classroom, such that the social distancing measures imposed by the state can be better complied with. In the case of *mirror classrooms*, they overcome many of the difficulties that the digital divide can cause (Beatty, 2019; Binnewies and Wang, 2019). Although the interest of this cited study focuses on undergraduate teaching, it is necessary to carry out the same study with other University stages. Other investigators have shown that the *blended learning training modality* in postgraduate programs is currently in high demand in Spain, and for this reason, the academic institutions promote their programs through the Internet, intending to attract students and promote the quality of their programs and institutions (López Catalán et al., 2018). According to Goodyear and other researchers, most studies highlight that hybrid learning modifies the role played by students. They take part not only as participants but also as protagonists in their learning, even becoming the co-configurators of learning environment/activities together with other apprentices (Beatty, 2019; Goodyear, 2020; Raes et al., 2020).

However, we acknowledged that there is considerable discussion among researchers regarding the disadvantages of *Hybrid learning*. The results of blended education in 2021 are worse in the public University (Figure 4, Items 1–3) due to the technical problems experienced by students during their development. If the technical means are not very good, i.e., if there are failures in the cameras, the microphone, or the program used by students to connect at home, students will lose their attention and interest. If certain technical requirements are not met, it will not be an effective pedagogical method (Hwang, 2018; Simpson, 2018; Traxler, 2018; Leoste et al., 2019; Goodyear, 2020; Hod and Katz, 2020).

In this study, we found differences between universities depending on the economic investment they had made in their facilities. In the case of the public University, the resources needed to broadcast the class from the University classroom for the blended methodology were scarce. There was no camera installed in the classroom, so the class had to be broadcast through the camera of a portable device, laptop, tablet, mobile, etc., in such a way that only one part of the class could be seen at one time, i.e., the face of the teacher or the blackboard. The private University installed high-quality cameras and microphones so that the classes could be broadcast live. The teacher just had to turn on the camera and connect to the Blackboard platform. The process was very simple. The students assessed that the quality of the media was adequate to fully follow the class from home.

There is no previous research using the *Online guide classroom* approach. To our knowledge, the *Online guide classroom* is an innovative methodology, appropriate for the times we live in, which potentially offers many advantages in the various situations studied in this research. It can be especially advantageous for confined situations, in science subjects where students need to use the laboratory, or under meteorological circumstances that make it impossible to travel to class. However, it also has shortcomings, as can occur with other blended methodologies, i.e., failures in technical infrastructure, image quality, audio, internet connection, etc., so universities need to make a stronger investment in technology. In addition, it will be necessary to have a person in charge of connecting devices and solving technical problems. This type of methodology, from our own experience, works very well in classes where students are autonomous and respectful, but it could be more complicated with less involved students.

## Conclusion

One of the direct consequences of the pandemic has been the need, for both teachers and students, to modify teaching methodologies. During the periods in which the cumulative incidence of COVID-19 was at very high levels, it was necessary to engage in distance education or blended education, and teachers had to quickly readjust to these changes. This adaptation has meant a great enrichment of their knowledge in new didactic resources to teach their classes.

Thanks to training during the *distance education* and *blended education* classes, face-to-face teachers have learned new skills. As a result of the enrichment of their knowledge in new didactic resources, the innovation in teaching methodologies and the ICT knowledge of teachers improved due to the pandemic. In this study, Item 6, i.e., ICT Knowledge, improved 3.2 pp, and Item 5, i.e., Innovative value of teaching practice, was also better valued in 2021 because teachers learned new applications and methodologies for online and hybrid teaching that they later incorporated into face-to-face classes.

Other important aspects to consider are accessibility, participation, and satisfaction, i.e., Items 1, 2, and 3, which also improved substantially in Study 2. There was a 1.2-pp increase in satisfaction with the *presence learning* in Study 2 compared with Study 1. The students in Study 2, who had already experienced the restrictions and social distancing due to the pandemic, valued these items much more than the students in Study 1, which is very likely because they were able to enjoy face-to-face teaching again. Those aspects that one does not notice on a day-to-day basis may have begun to become more important once they were lost.

These results provide a basis to carefully consider this new era in higher education. We assumed that it is essential to change the paradigm of in-University education. Any subject—understood as an educational subject—should not be approached as a body of finished knowledge but as living knowledge that can be transmitted in person or not, depending on whether the context allows it. Similarly, it will be essential that teachers have enough ICT training to be able to use the most appropriate software and adapt to other pandemics that may befall society so that they can continue to provide quality education to students. It is not a problem of the satisfaction with different formats, be in-person, online, or a combination, as we have discussed previously, although most of the students state that they prefer a face-to-face format due to the interaction with their classmates, better participation in the classroom, and better understanding of the teacher. To be able to adequately follow daily teachings, there is indeed a need for the students themselves to have the necessary resources at their disposal, such as Internet connection and smart devices, so that they can connect to classes that today seem accessible to any University student of a developed country, but it will be important to provide the necessary resources for all students of any social context. Based on our experience, we supported the idea that, working together with educational centers, mixed teaching, or remote teaching are the best facilitators of learning, and neither COVID-19 nor any other pandemic can stop teaching if we have the knowledge, skills, and adequate resources.

The literature highlights deficiencies in the transition from face-to-face teaching to online teaching due to the weakness of the online teaching infrastructure, the inexperience of teachers, the digital divide, a complex home environment, etc. Although the advances in the use of educational technology support remote learning since the pandemic became a reality, the efforts to use this technology, and the large-scale advances in the distance and online education during the pandemic compel us to face an unprecedented technological revolution and take advantage of it. It is important to highlight the fact that, if the pandemic had not had this impact on education, the use of the technological evolution in educational environments might not have evolved so quickly in the last few months.

The results of the experiment show clear support for the *presence learning* from students in the public University, as they appeared to be more satisfied with it than with any of the other methodologies. The public University students preferred the *online teaching* as a second option since the quality of the class was also very high, and the *blended learning* was preferred the least. The satisfaction of the private University students was very similar with respect to these three teaching methods used in this University, with a slightly higher preference for face-to-face teaching.

This research provides new perspectives on a category of teaching education in the period of COVID-19. The results obtained corroborate conclusions reached by other studies (Hwang, 2018; Leoste et al., 2019; Goodyear, 2020; Wang et al., 2020), in which learning can be carried out in an efficient, high-quality, and satisfactory manner either through methods we choose or through methods to which the context forces us to adapt. We must choose a modality without affecting education.

Future investigations can validate the conclusions drawn from this study. In this study, we described preferences of teaching and learning modalities and showed that, although students value the possibilities of technology very positively, face-to-face communication with the teacher, to a larger degree, is believed to be required for success in their studies.

## Data Availability Statement

The original contributions presented in the study are included in the article/supplementary material, further inquiries can be directed to the corresponding author/s.

## Ethics Statement

Ethical approval was not required for this study in line with local legislation and institutional requirements. Before administering the questionnaire to the students, compliance with all the required ethical standards was ensured as follows: written informed consent, the right to information, confidentiality, anonymity, gratuity, and the option to abandon the study.

## Author Contributions

AV and JMV conceptualized and wrote the manuscript. Both authors contributed to the article and approved the submitted version.

## Conflict of Interest

The authors declare that the research was conducted in the absence of any commercial or financial relationships that could be construed as a potential conflict of interest.

## Publisher's Note

All claims expressed in this article are solely those of the authors and do not necessarily represent those of their affiliated organizations, or those of the publisher, the editors and the reviewers. Any product that may be evaluated in this article, or claim that may be made by its manufacturer, is not guaranteed or endorsed by the publisher.
